# Synopsis of the aurantiactinomyxon collective group (Cnidaria, Myxozoa), with a discussion on the validity of morphotype definition and demise of guyenotia

**DOI:** 10.1007/s11230-023-10089-1

**Published:** 2023-04-15

**Authors:** Sónia Rocha

**Affiliations:** 1 Instituto de Investigação e Inovação em Saúde (i3S), University of Porto, Rua Alfredo Allen no. 208, 4200-135 Porto, Portugal; 2 ICBAS–School of Medicine and Biomedical Sciences, University of Porto, Rua Jorge Viterbo Ferreira no. 228, 4050-313 Porto, Portugal

## Abstract

Aurantiactinomyxon is one of the most diverse myxozoan collective groups, comprising types that mostly infect freshwater and marine oligochaetes belonging to the family Naididae Ehrenberg, 1828, but also Lumbriculidae Claus, 1872. In this study, a comprehensive revision of all known aurantiactinomyxon types is performed and highlights the fallibility of using the form and length of the valvular processes as main criterion for differentiating among style-less actinospore morphotypes. The demise of the guyenotia collective group is proposed based on the ambiguous features of several types that allow conformity with both the aurantiactinomyxon and guyenotia definitions. Nonetheless, the information presently available clearly shows that a general shift is needed in our approach to actinospore grouping, which should probably be based on actinospore functionality relative to environment and host ecology, rather than on morphology. Life cycle studies based on experimental transmission and molecular inferences of the 18S rDNA have linked aurantiactinomyxon (including former guyenotia) to myxozoans belonging to a diverse array of genera, including *Chloromyxum*, *Henneguya*, *Hoferellus*, *Myxobolus*, *Paramyxidium*, *Thelohanellus* and *Zschokkella*. This undoubtedly shows a high capacity of the aurantiactinomyxon morphotype to promote infection in intrinsically distinct vertebrate hosts and environmental habitats, consequently increasing interest in its study for attaining a better understanding of myxozoan-host interactions. The identification of novel and known types, however, is impeded by the lack of concise information allowing a comprehensive analysis of biological, morphological, and molecular criteria. In this sense, the compilation of data presented in this study will ultimately help researchers seeking to perform reliable identifications.

## Introduction

Infections of aquatic oligochaetes by actinospores were first reported by Štolc (1899), who created the Actinomyxidia to encompass hexactinomyxon, synactinomyxon and triactinomyxon types that the author found infecting tubificids in Czechia. Over time, recognition of the homology between these organisms and myxozoans, led to the relocation of Actinomyxidia to the phylum Myxozoa Grassé, 1970, which became divided into the classes Myxosporea Bütschli 1881 (fish parasites producing myxospores) and Actinosporea Noble, 1980 (worm parasites producing actinospores). In 1984, the ground-breaking discovery that *Myxobolus cerebralis* Hofer, 1903 develops triactinomyxon actinospores in the gut epithelium of the oligochaete *Tubifex tubifex* (Müller) (Wolf & Markiw, 1984), showed that myxozoan life cycles comprise both myxospore and actinospore phases, with members of the classes Myxosporea and Actinosporea representing morphologically distinct phases of the same species. This led to a major taxonomic revision, with Kent et al. (1994) proposing the demise of the class Actinosporea, and the use of its generic names as vernacular designations for actinospore morphotypes established within distinct collective groups.

Currently, there are ca. 20 valid actinospore collective groups (Lom & Dyková, 2006; Rangel et al., 2011; Milanin et al., 2017; Atkinson et al., 2019; Rocha et al., 2019a, 2019b, 2020), with aurantiactinomyxon being one of the most diverse. This collective group was first described by Janiszewska (1957), who defined its actinospores having a style-less epispore, with three equal processes that curve downwards and embrace with their whole base the epispore cavity. Lom & Dyková (2006) updated the definition, and described aurantiactinomyxon as having three stout, semicircularly curved, leaf-like valvular processes attached to an ellipsoidal body with protruding polar capsules at the apex and containing a sporoplasm with many secondary cells. To date, 61 aurantiactinomyxon types have been reported based on these definitions (Table 1), with differentiation between types mostly relying on morphometric comparisons. Molecular data of the 18S rDNA is available for only 20 types. Another eight 18S rDNA sequences are available in GenBank but constitute unpublished submissions to the NCBI database; while the sequences with GenBank accession numbers MN294775 and MN294776 appear identified as aurantiactinomyxon in the database but have been published as belonging to the presently demised echinactinomyxon collective group (see Rocha et al., 2019a; Gao et al., 2021).

**Table 1:** Summary of data available for aurantiactinomyxon types (including former guyenotia). SBL: actinospore body length; SBW: actinospore body width; LVP: length of valvular processes; WVP: width of valvular processes; PCL: polar capsule length; PCW: polar capsule width; SCn: number of secondary cells; AV: apical view; SV: side view; n.d.: not provided; ET: experimental transmission; MI: molecular inference. Measurements are means ± SD (range) (when available), given in µm.

Aurantiactinomyxon type/myxosporean species	Host	Location	SBL	SBW	LVP	WVP	PCL	PCW	SCn	GenBank accession number	Source
Aurantiactinomyxon raabei junioris Janiszewska, 1957	*Limnodrilus hoffmeisteri* Claparède	Poland: River Ropa	17	17	25–30	−	−	−	16	−	Janiszewska 1957
	*Limnodrilus* sp.; *Tubifex* sp.	France: Villeneuve de la Raho, Latour-Bas-Elne, Bages, Roussillon, Pyrénées-Orientales	17	17	25–35	11–15	−	−	16	−	Marques, 1984
Aurantiactinomyxon pavinsis (Ormierès, 1968) Marques, 1984/*Chloromyxum truttae* (Léger, 1906)/MI	*Stylodrylus heringianus* Claparède	France: Besse-en-Chandesse	10 (9–11)	10 (9–11)	12 (10–14)	−	3.0 (2.5–3.5)	2.0 (1.5–2.5)	12	−	Ormières, 1968
	*Stylodrylus heringianus* Claparède	France: Couze Pavin	8–12	8–12	10–20	6–8	−	−	16	−	Marques, 1984
	*Tubifex* sp.	Germany: Landsberg am Lech, Bavaria	10.0 ± 1.0	10.0 ± 1.0	12.0 ± 2.0	−	3.0 ± 0.5	2.0 ± 0.5	12	−	Oumouna et al., 2003
	*Stylodrylus heringianus* Claparède	Scotland: highland freshwater system of the River Amhainnan Stratha Bhig	9.0	9.0	10.0	−	2.8	2.3	−	AJ582006	Holzer et al., 2004
	Unidentified lumbriculid	Italy: Sordo River	9.7 ± 0.5	9.7 ± 0.5	17.4 ± 1.4	7.7 ± 0.5	−	−	−	−	Marcucci et al.,2009
Aurantiactinomyxon stellans Marques, 1984	Unidentified tubificids	France: Villeneuve de la Raho, Bages, Plaine du Roussillon, Pyrénées-Orientales	15–20	–	70–90	15–20	8–10	8–10	<16	–	Marques, 1984
Aurantiactinomyxon trifolium Marques, 1984	Unidentified tubificids	France: Villeneuve de la Raho, Latour-Bas-Elne, Bages, Roussillon, Pyrénées-Orientales	20–25	20–25	40–50	17–20	–	–	32	–	Marques, 1984
Aurantiactinomyxon of Burtle et al., 1991/*Henneguya ictaluri* Pote, Hanson, & Shivaji, 2000/ET and MI^a^	*Dero digitata* (Müller)	USA: channel catfish pond	19.5	19.5	29.5	–	–	–	>32	–	Burtle et al., 1991;Bellerud et al., 1992, 1995; Pote et al., 1992, 2000; Styer et al., 1991, 1992
	*Dero digitata* (Müller)	USA: channel catfish pond, Oktibbeha County, Mississippi	20.6 ± 1.1 (19.0–22.8)	21.8 ± 1.0 (20.9–22.8)	28.4 ± 1.6 (26.6–32.3)	9.9 ± 0.8 (9.5–11.4)	–	–	40–42	–	Bellerud, 1993
	*Dero digitata* (Müller)	USA: channel catfish pond, Oktibbeha County, Mississippi	23.0 (20.0–24.0)	23.0 (20.0–24.0)	26.0 (21.0–32.0)	11.0 (8.0–12.0)	–	–	–	–	Pote & Waterstrat, 1993
	*Dero digitata* (Müller)	USA: channel catfish pond, Sunflower County, Mississippi	20.9 ± 0.6 (20.1–22.3)	20.9 ± 0.6 (20.1–22.3)	27.7 ± 0.7 (26.3–28.9)	10.0 ± 0.4 (9.4–10.9)	–	–	–	–	Rosser et al., 2014
Aurantiactinomyxon minor Styer et al., 1992	*Dero digitata* (Müller)	USA: channel catfish pond	13–16	13–16	36	11	–	–	–	–	Styer et al., 1992
	*Limnodrilus hoffmeisteri* Claparède	Ireland: Cloonee river system	14.1 ± 1.3 (13.0–15.6)	14.1 ± 1.3 (13.0–15.6)	31.0 ± 3.7 (26.0–36.0)	10.6 ± 1.1 (9.1–13.0)	2.7 ± 0.2 (2.6–3.1)	2.7 ± 0.2 (2.6–3.1)	~12	–	Negredo & Mulcahy, 2001
Aurantiactinomyxon type 1 of Bartholomew et al., 1992	*Nais bretscheri* Michaelsen	USA: experimental tanks	–	–	–	–	–	–	–	–	Bartholomew et al., 1992
Aurantiactinomyxon of El-Matbouli et al., 1992/*Hoferellus carassii* Achmerov, 1960/ET	*Tubifex tubifex* (Müller), *Lophochaeta ignota* Štolc, *Limnodrilus hoffmeisteri* Claparède	ET using infected goldfish *Carassius auratus*	–	–	–	–	–	–	–	–	El-Matbouli et al., 1992
	*Nais elinguis* Müller	Germany	23.5 ± 3.1	23.5 ± 3.1	48.8 ± 8.2	11.7 ± 1.5	–	––	22	–	Trouillier et al., 1996
Aurantiactinomyxon of Grossheider & Korting, 1992/*Hoferellus cyprini* (Doflein, 1898) Berg, 1898 (syn. *Mitraspora cyprini* Fujita, 1912)/ET^b^	*Nais* spp.	Germany: ET using *H. cyprini* from common carp *Cyprinus carpio*	~12.7	~12.7	~31.1	~6.9	~6.9	–	–	–	Grossheider & Korting, 1992
Aurantiactinomyxon janiszewskai Bellerud, 1993/*Henneguya exilis* (Kudo, 1929)/MI	*Dero digitata* (Müller)	USA: Sunflower, Sunflower County, Mississippi	13.8 ± 1.1 (11.8–15.8)	11.8 ± 1.1 (9.9–13.8)	52.0 ± 9.9 (37.4–63.0)	5.9 ± 0.0 (5.9–5.9)	–	–	8	–	Bellerud, 1993
	*Dero digitata* (Müller)	USA: commercial catfish ponds, Mississippi	–	–	–	–	–	–	–	–	Lin et al., 1999
	*Dero digitata* (Müller)	USA: commercial catfish pond, Sunflower County, Mississippi	11.7 ± 0.9 (10.2–13.3)	11.7 ± 0.9 (10.2–13.3)	42.5 ± 2.5 (37.6–46.2)	6.5 ± 0.9 (5.2–8.5)	–	–	–	–	Rosser et al., 2014
Aurantiactinomyxon mississippiensis Bellerud, 1993/*Henneguya mississippiensis* Rosser et al., 2005/MI	*Dero digitata* (Müller)	USA: Starkville, Oktibbeha County, Mississippi	14.2 ± 1.8 (n.d.–17.1)	13.6 ± 1.2 (9.9–13.8)	32.4 ± 3.3 (22.8–39.9)	7.3 ± 1.7 (3.8–11.4)	–	–	30	−	Bellerud, 1993
	*Dero digitata* (Müller)	USA: channel catfish pond	–	–	–	–	–	–	–	AF021878	Hanson et al., 2001
Aurantiactinomyxon of Benajiba & Marques, 1993/*Paramyxidium giardi* (Cépède, 1906) Freeman & Kristmundsson, 2018/ET and MI	*Tubifex* spp.	France: ET using *Paramyxidium giardi* from European eel *Anguilla anguilla*	–	–	–	–	–	–	–	–	Benajiba & Marques, 1993
	*Tubificoides pseudogaster* (Dahl)	Portugal: Minho River	14.4 ± 0.6 (13.6–15.9)	12.7 ± 0.7 (11.3–13.3)	22.4 ± 2.4 (18.1–27.6)	15.5 ± 0.9 (13.3–17.0)	2.6 ± 0.3 (1.9–3.5)	2.6 ± 0.3 (1.9–3.5)	–	MK635346	Rocha et al., 2019c
Aurantiactinomyxon of Pote & Waterstrat, 1993	*Dero digitata* (Müller)	USA: channel catfish pond, Oktibbeha County, Mississippi	~23.0	~23.0	40	8	–	–	–	–	Pote & Waterstrat, 1993
Aurantiactinomyxon type 1 of Yokoyama et al., 1993	*Branchiura sowerbyi* Beddard	Japan: goldfish *Carassius auratus* pond	11	11	16	–	–	–	8	–	Yokoyama et al., 1993; Yokoyama, 1997
Aurantiactinomyxon type 2 of Yokoyama et al., 1993/*Thelohanellus hovorkai* Achmerov, 1964/ET and MI	*Branchiura sowerbyi* Beddard	Japan: goldfish *Carassius auratus* pond	–	–	–	–	–	–	–	–	Yokoyama et al., 1993
	*Branchiura sowerbyi* Beddard	Japan: common carp *Cyprinius carpio* fry pond	18–22	18–22	25–33	–	2	2	32	–	Yokoyama, 1997
	*Branchiura sowerbyi* Beddard	Hungary: ET using *Thelohanellus hovorkai* from a *C. carpio* fish farm	18.6 (18.3–18.9)	18.6 (18.3–18.9)	29.0 (28.2–29.6)	9.2 (8.1–10.2)	3.42 (3.4–3.5)	3.36 (3.3–3.4)	32	−	Székely et al., 1998
	*Branchiura sowerbyi* Beddard	Japan	–	–	–	–	–	–	–	AJ133419	Anderson et al., 2000
Aurantiactinomyxon type 1 of Hallett et al., 1997	*Pacifidrilus vanus* (Erséus)	Hong Kong Island	10.1	10.7	~3.0	~3.0	1.9	1.9	−	−	Hallett et al., 1997
Auratiactinomyxon type 2 of Hallett et al., 1997	*Pacifidrilus darvelli* (Erséus) *Limnodriloides toloensis* Erséus	Hong Kong Island	9.4–12.5	11.6–14.0	–	–	2.5–3.0	2.5–3.0	−−	−	Hallett et al., 1997
Aurantiactinomyxon type 3 of Hallett et al., 1997	*Pacifidrilus vanus* (Erséus)	Hong Kong Island	9.4–10.6	6.9–10.9	–	−	−	−−	−	−−	Hallett et al., 1997
Aurantiactinomyxon of McGeorge et al., 1997	Unidentified tubificid	Scotland: Atlantic salmon *Salmo salar* hatchery	13.7 (12–15)	13.7 (12–15)	25.6 (19–31)	12.0 (10–14)	2.7 (2–3)	2.7 (2–3)	−	−	McGeorge et al., 1997
	*Lumbriculus variegatus* (Müller)	Scotland: Atlantic salmon *Salmo salar* hatchery	–	–	–	–	–	–	–	–	Özer & Wootten, 2001
Aurantiactinomyxon type 1 of El-Mansy et al., 1998a	*Tubifex tubifex* (Müller)	Hungary: polyculture fish farm south of Budapest	18.3	18.3	17.5	9.9	2.0	2.0	–	–	El-Mansy et al., 1998a
Aurantiactinomyxon type 2 of El-Mansy et al., 1998a	*Branchiura sowerbyi* Beddard	Hungary: polyculture fish farm south of Budapest	22.8	22.8	65.7	10.5	4.0	1.7	–	–	El-Mansy et al., 1998a
Aurantiactinomyxon type 3 of El-Mansy et al., 1998a	*Branchiura sowerbyi* Beddard	Hungary: polyculture fish farm south of Budapest	22.8	22.8	70.3	8.0	2.9	2.9	–	–	El-Mansy et al., 1998a
Aurantiactinomyxon type 4 of El-Mansy et al., 1998a	*Branchiura sowerbyi* Beddard	Hungary: polyculture fish farm south of Budapest	19.4	19.4	55.7	11.2	2.9	2.9	–	–	El-Mansy et al., 1998a
Aurantiactinomyxon type 5 of El-Mansy et al., 1998a	*Branchiura sowerbyi* Beddard	Hungary: polyculture fish farm south of Budapest	9.9	9.9	17.2	3.9	1.4	1.4	–	–	El-Mansy et al., 1998a
Aurantiactinomyxon type 6 of El-Mansy et al., 1998a	*Limnodrilus* sp.	Hungary: polyculture fish farm south of Budapest	19.7	19.7	24.2	11.2	2.8	2.8	–	–	El-Mansy et al., 1998a
Aurantiactinomyxon type 7 of El-Mansy et al., 1998a	Actinospores collected from water	Hungary: polyculture fish farm south of Budapest	18.9	18.9	24.4	9.5	2.8	2.5	–	–	El-Mansy et al., 1998a
Aurantiactinomyxon type 8 of El-Mansy et al., 1998a	*Limnodrilus* sp.	Hungary: polyculture fish farm south of Budapest	22.6	22.6	12.2	9.0	1.4	1.4	−	−	El-Mansy et al., 1998a
Aurantiactinomyxon type 9 of El-Mansy et al., 1998a	*Branchiura sowerbyi* Beddard	Hungary: polyculture fish farm south of Budapest	18.8	18.8	51.3	9.5	2.3	2.3	−	−	El-Mansy et al., 1998a
Aurantiactinomyxon type 10 of El-Mansy et al., 1998a	*Branchiura sowerbyi* Beddard	Hungary: polyculture fish farm south of Budapest	15.5	15.5	16.7	8.8	1.7	1.7	–	–	El-Mansy et al., 1998a
Aurantiactinomyxon type 11 of El-Mansy et al., 1998a	Actinospores collected from water	Hungary: polyculture fish farm south of Budapest	8.5	8.5	31.9	3.7	3.4	2.0	–	–	El-Mansy et al., 1998a
Aurantiactinomyxon type 12 of El-Mansy et al., 1998a	*Branchiura sowerbyi* Beddard	Hungary: polyculture fish farm south of Budapest	12.1	12.1	26.5	8.7	2.8	3.1	–	–	El-Mansy et al., 1998a
Aurantiactinomyxon type 1 of El-Mansy et al., 1998b	*Branchiura sowerbyi* Beddard	Hungary: Lake Balaton	18.8	18.8	51.3	9.5	2.3	2.3	–	–	El-Mansy et al., 1998b
Aurantiactinomyxon type 2 of El-Mansy et al., 1998b	*Limnodrilus* sp.	Hungary: Lake Balaton	21.1	21.1	22.6	11.7	2.8	2.0	–	–	El-Mansy et al., 1998b
Aurantiactinomyxon type 3 of El-Mansy et al., 1998b	*Branchiura sowerbyi* Beddard	Hungary: Lake Balaton	9.9	9.9	17.2	3.9	1.4	1.4	–	–	El-Mansy et al., 1998b
Aurantiactinomyxon of Székely et al., 1998/*Thelohanellus nikolskii* Achmerov, 1955/ET and MI	*Tubifex tubifex* (Müller)	Hungary: ET using *T. nikolskii* from infected *Cyprinus carpio* in the Kis-Balaton reservoir	21.1 (21.0–21.2)	21.1 (21.0–21.2)	13.4 (11.3–15.5)	9.0 (8.5–9.6)	2.1 (2.0–2.2)	2.1 (2.0–2.2)	16	–	Székely et al., 1998
	*Nais* sp.	Hungary: Kis Balaton	10.3 (9.3–12.0)	10.3 (9.3–12.0)	14.6 (12.7–16.0)	6.5 (5.3–7.3)	3.3	2.6	8	DQ231156	Borkhanuddin, 2013; Borzák et al., 2021
Aurantiactinomyxon of Xiao & Desser, 1998	*Limnodrilus hoffmeisteri* Claparède	Canada: Lake Sasajewun, Ontario	12.0 (11.5–13.8)	11.0 (10.0–12.5)	24.0 (21.0–26.0)	13.0–16.0	3.0 (2.7–3.4)	1.5 (1.4–1.7)	64–128	−	Xiao & Desser, 1998
Aurantiactinomyxon of Székely et al., 2000	*Branchiura sowerbyi* Beddard	Spain: Mijares River, Province of Castellón	8.1 (7.3–8.9)	8.1 (7.3–8.9)	6.1 (4.8–7.3)	5.6 (4.8–6.5)	1.6 (1.5–1.7)	1.1 (1.0–1.1)	64	−−	Székely et al., 2000
Aurantiactinomyxon of Kent et al., 2001	*Limnodrilus hoffmeisteri* Claparède	Canada: Ontario	–	–	–	–	–	–	–	AF378356	AF378356 Kent et al., 2001
Aurantiactinomyxon type A1 of Negredo & Mulcahy, 2001^c^	*Lophochaeta ignota* Štolc	Ireland: Cloonee river system	14.4 ± 1.3 (12.6–16.9)	14.4 ± 1.3 (12.6–16.9)	21.1 ± 1.0 (18.2–23.4)	16.1 ± 2.1 (13.0–19.5)	3.0 ± 0.3 (2.5–3.9)	3.0 ± 0.3 (2.5–3.9)	10	–	Negredo & Mulcahy, 2001
	*Lophochaeta ignota* Štolc	Ireland: Cloonee river system	–	–	–	–	–	–	–	AF483598	Negredo et al., 2003
Aurantiactinomyxon type A3 of Negredo & Mulcahy, 2001	*Lophochaeta ignota* Štolc	Ireland: Cloonee river system	~9.1	~9.1	20.8 ± 1.8 (18.2–23.4)	10.4	–	–	~10	~	Negredo & Mulcahy, 2001
Aurantiactinomyxon of Hallett et al., 2002^d^	*Tubifex tubifex* (Müller)	Germany: Bavaria	19.4 (16.8–21.4)	19.4 (16.8–21.4)	37.3 (28.5–49.2)	15.7 (14.2–18.1)	3.1 (2.6–3.9)	3.1 (2.6–3.9)	30	AF487455	Hallett et al., 2002
	*Tubifex tubifex* (Müller)	Germany: Bavaria	19.7 (18.1–22.0)	19.7 (18.1–22.0)	87.7 (75.1–103.6)	13.1 (10.4–15.5)	3.1	3.1	30	AF487455	Hallett et al., 2002
Aurantiactinomyxon type 1 of Özer et al., 2002	*Tubifex tubifex* (Müller)	Scotland: Atlantic salmon fish farm	14.4 (12.0–15.0)	14.4 (12.0–15.0)	32.0 (31.0–36.0)	14.8 (13.0–15.0)	2.7 (2.0–3.0)	2.7 (2.0–3.0)	64–128	–	Özer et al., 2002a
	*Tubifex tubifex* (Müller)	Scotland: Atlantic salmon fish farm	14.2	14.2	33.0	–	2.6	2.5	−	AJ582004	Holzer et al., 2004
Aurantiactinomyxon type 2 of Özer et al., 2002	*Tubifex tubifex* (Müller)	Scotland: Atlantic salmon fish farm	14.9 (14.0–18.7)	14.9 (14.0–18.7)	24.8 (23.4–26.5)	15.3 (14.0–15.6)	2.5 (1.8–2.8)	2.5 (1.8–2.8)	64	−	Özer et al., 2002a
Aurantiactinomyxon type 3 of Özer et al., 2002	*Tubifex tubifex* (Müller)	Scotland: Atlantic salmon fish farm	24.0 (23.4–24.9)	21.8 (20.8–23.4)	114.5 (101.4–124.8)	–	4.0	3.2	32	–	Özer et al., 2002a
	*Tubifex tubifex* (Müller)	Scotland: Atlantic salmon fish farm	21.1	19.3	114.0	–	4.0	3.5	–	AJ582005	Holzer et al., 2004
Aurantiactinomyxon type 4 of Özer et al., 2002	*Tubifex tubifex* (Müller)	Scotland: Atlantic salmon fish farm	11.9 (11.2–14.0)	11.9 (11.2–14.0)	28.3 (23.4–31.2)	11.9 (10.9–14.0)	2.5 (2.0–3.0)	2.5 (2.0–3.0)	32	–	Özer et al., 2002a
Aurantiactinomyxon type 1 of Oumouna et al., 2003	Unidentified	Gremany: trout fish farm, Landsberg am Lech, Bavaria	16.1 ± 1.0	16.1 ± 1.0	76.0 ± 1.0	–	5.0 ± 0.3	4.0 ± 0.2	–	–	Oumouna et al., 2003
Aurantiactinomyxon type 1 of Székely et al., 2003	*Tubifex tubifex* (Müller)	Japan: Fuji Mountain at Honshu	13.5 (13.0–14.0)	13.5 (13.0–14.0)	12.4 (10.0–14.0)	13.5 (13.0–14.0)	2.0	1.0	8	–	Székely et al., 2003
Aurantiactinomyxon of Székely et al., 2004	*Branchiura sowerbyi* Beddard	South Africa: Rietvlei River	19.6 (18.1–21.8)	19.6 (18.1–21.8)	10.5 (9.9–17.4)	15.2 (13.3–18.7)	2.7 (2.6–2.9)	2.7 (2.6–2.9)	64	−	Székely et al., 2004
Aurantiactinomyxon type A of Eszterbauer et al., 2006/*Thelohanellus hovorkai* Achmerov, 1964/ET and MI	*Branchiura sowerbyi* Beddard	Hungary: fish farm near Budapest	20.0 (18.0–22.0)	20.0 (18.0–22.0)	47.0 (37.0–58.0)	10.0 (8.0–12.0)	3.0 (2.0–3.3)	–	32	DQ231153 DQ231154	Eszterbauer et al., 2006
Aurantiactinomyxon type B of Eszterbauer et al., 2006^d^	*Branchiura sowerbyi* Beddard	Hungary: fish farm near Budapest	18.0 (17.0–20.0)	18.0 (17.0–20.0)	24.0 (20.0–30.0)	9.8 (9.0–10.0)	2.5 (2.0–3.0)	–	–	–	Eszterbauer et al., 2006
	*Branchiura sowerbyi* Beddard	Hungary: River Tisza	19.0 (18.0–21.0)	19.0 (18.0–21.0)	16.0 (14.0–20.0)	8.4 (7.0–10.6)	2.6 (2.0–4.1)	−	−	DQ231148	Eszterbauer et al., 2006
Aurantiactinomyxon of Hallett et al., 2006/*Myxobolus intimus* Zaika, 1965/MI	*Limnodrilus hoffmeisteri* Claparède	Germany: pet shop in Munich	AV 13.8 (13.0–14.9) SV 13.6	AV 13.8 (13.0–14.9) SV 13.6	AV 17.7 (15.5–22.0) SV 20.1 (18.1–22.0)	AV 10.7 (9.7–14.2) SV 10.4	3.1	2.3	16	AY495708	Hallett et al., 2006
Aurantiactinomyxon type 1 of Hallett et al., 2006	Unidentified	Germany: pet shop in Munich	AV 12.0 (11.7–13.0)	AV 12.0 (11.7–13.0)	AV 26.6 (24.6–31.1)	AV 10.1 (9.1–10.4)	2.3	3.0	16	−	Hallett et al., 2006
Aurantiactinomyxon of Morris & Freeman, 2010	*Tubifex tubifex* (Müller)	Scotland: brown trout *Salmo trutta* fish farm	11.0 ± 1.0	11.0 ± 1.0	23.0 ± 4.0 (20.0–33.0)	11.0 ± 1.0 (11.0–13.0)	−	−	~16	−	Morris & Freeman, 2010
Aurantiactinomyxon of Xi et al., 2013	*Branchiura sowerbyi* Beddard	China: crucian carp pond, Jiangsu Province	AV 19.7 (18.9–21.1) SV 20.3 (18.6–21.9)	AV 19.7 (18.9–21.1) SV 19.6 (17.8–20.7)	170.8 (167.5–176.3)	12.9 (11.2–13.5)	3.1 (2.9–3.2)	1.7 (1.5–1.9)	64	HQ613406	Xi et al., 2013
Aurantiactinomyxon type JD of Xi et al., 2015	*Branchiura sowerbyi* Beddard	China: fish farm at Jiangsu Province	15.6	21.2 (17.1–24.0)	21.7 (20.0–24.4)	14.0 (11.2–16.4)	2.3 (2.0–2.8)	2.3 (2.0–2.8)	>30	KP642133– 34	Xi et al., 2015
Aurantiactinomyxon types 1 & 2 of Zhao et al., 2016/*Thelohanellus kitauei* Egusa & Nakajima, 1981/MI^d,e^	*Branchiura sowerbyi* Beddard	Hungary: Kis-Balaton Reservoir	19.7 (17.3–23.3)	19.7 (17.3–23.3)	20.4 (18.7–23.3)	8.9 (7.4–10.0)	3.4	2.8	>28	KU66464	Zhao et al., 2016
	*Branchiura sowerbyi* Beddard	China: Datong Lake, Honghu City, Hubei Province	AV 20.9 (19.3–22.1) SV 18.4 (17.6–23.0)	AV 20.9 (19.3–22.1) SV 22.3 (21.6–23.0)	19.7 (17.9–22.3)	11.6 (9.8–13.0)	3.0 (2.8–3.3)	2.4 (2.2–2.6)	32	KU664644	Zhao et al., 2016
Aurantiactinomyxon of Zhao et al., 2017/*Thelohanellus testudineus* Liu et al., 2013/MI	*Branchiura sowerbyi* Beddard	China: *Carassius auratus gibelio* fish farm, Hubei Province	15.5 ± 0.5 (14.5–16.4)	15.5 ± 0.5 (14.5–16.4)	13.2 ± 0.9 (11.5–16.2)	7.4 ± 0.4 (6.7–8.0)	2.5 ± 0.2 (2.3–2.9)	2.0 ± 0.2 (1.8–2.4)	32	KY475588 KY475589	Zhao et al., 2017
Aurantiactinomyxon of Milanin et al., 2017	*Pristina americana* Černosvitov	Brazil: fish farm in Porto Ferreira city, São Paulo State	10.9 (8.7–11.5)	10.9 (8.7–11.5)	18.6 (18.1–20.1)	9.0 (8.5–9.4)	2.1 (1.6–2.4)	2.1 (1.6–2.4)	−	KX068207	Milanin et al., 2017
Aurantiactinomyxon of Freeman & Kristmundsson, 2018	Lumbriculus variegatus (Müller)	Iceland: lake Vifilsstadavatn	10.4 (9.0–12.5)	10.4 (9.0–12.5)	15.4 (14.5–16.3)	8.5 (7.5–10.0)	=	=	=	MH414928	Freeman & Kristmundsson (2018)
Aurantiactinomyxon type 1 of Milanin et al., 2018	*Pristina synclites* Stephenson	Brazil: fish farm in Terenos city, Mato Grosso do Sul state	8.7 (8.2–9.7)	8.7 (8.2–9.7)	14.6 (12.2–16.3)	6.9 (5.4–8.8)	1.3 (1.0–1.4)	1.3 (1.0–1.4)	−	MG878981	Milanin et al., 2018
Aurantiactinomyxon type 2 of Milanin et al., 2018	*Pristina synclites* Stephenson	Brazil: fish farm in Terenos city, Mato Grosso do Sul state	11.2 (10.2–11.9)	11.2 (10.2–11.9)	30.4 (25–33.3)	7.0(5.4–7.7)	1.5(1.3– 1.8)	1.5(1.3– 1.8)	−	MG878982	Milanin et al.,2018
Guyenotia sphaerulosa Naville, 1930	*Tubifex tubifex (Müller)*	France: Luc-sur-Mer, Calvados	15	15	40	−	6	5	32	−	Naville, 1930;Marques, 1984
Guyenotia of Xiao & Desser, 1998	*Lumbriculus variegatus (Müller)*	Canada: Lake Sasajewun, Ontario	9.5 (9.0–10.5)	8.8 (8.0–9.5)	21.0 (16.0–25.0)	4.5–6.5	3.0(2.8–3.3)	2.0(1.8–2.2)	8–16	−	Xiao & Desser,1998
Guyenotia of Eszterbauer et al., 2006	*Branchiura sowerbyi Beddard*	Hungary: Temperate Water Fish Farm near Budapest	10 (9.5–11)	10 (9.5–11)	16 (15–19.5)	4.5(4.0–5.5)	1.6(1.4–2.0)	−	−	AY77906	Eszterbauer et al.,2006
	*Branchiura sowerbyi Beddard*	Hungary: River Tisza near Tiszafüred	11 (10–12)	11 (10–12)	17 (15–21)	4.5(3.5–5.5)	1.6(1.3–2.0)	−	−	AY779063	Eszterbauer et al.,2006
Guyenotia of Xi et al., 2013	*Branchiura sowerbyi Beddard*	China: freshwater pond, Jiangsu	10.3 (9.6–10.9)	10.3 (9.6–10.9)	18.5 (16.5–20.6)	6.0(5.4–6.4)	−	1.8(1.6–2.1	−	−	Xi et al., 2013
Guyenotia type CZ of Sun et al., 2014	*Branchiura sowerbyi Beddard*	China: Changzhou	10.7 (10.0–12.1)	10.7 (10.0–12.1)	20.5 (17.4–23.8)	5.8(4.8–6.3)	1.8(1.7–2.0)	–	–	KF912953	Sun et al., 2014

^a^Described as Aurantiactinomyxon major by Styer et al. (1992). A 100% sequence similarity was reported by Rosser et al. (2014), but GenBank accession number was not provided.

^b^Measurements calculated from image available in the original description.

^c^Originally suggested to potentially be Aurantiactinomyxon pavinsis, but 18S rDNA does not match the sequence obtained by Holzer et al. (2004) from the type-host.

^d^Presents a case of polymorphism, in which two different phenotypes of Aurantiactinomyxon share one genotype.

^e^Type 1 was previously described as Aurantiactinomyxon type 4 by Borkhanuddin (2013), with slightly distinct measurements.

Assigning novel types to a specific collective group can be complicated given the overlapping definitions of several groups that share main morphological features, such as the formation of single spores versus multi-spore cages, and presence/absence of style or valvular processes. In fact, a boundary-less “continuum of form” has been suggested to exist between collective groups that differ based solely on the form of a specific morphological character, as is the case of aurantiactinomyxon, echinactinomyxon Janiszewska, 1957, guyenotia Naville, 1930, neoactinomyxum Granata, 1922 and raabeia Janiszewska, 1955 (see Hallett et al., 2006; Atkinson, 2011). Differentiation between these style-less morphotypes is based on the shape and length of the valvular processes, which are traditionally defined as being long and straight in echinactinomyxon, curved in raabeia, leaf-like and curved downwards in aurantiactinomyxon, digitiform in guyenotia, and short and spherical in neoactinomyxum (see review in Lom & Dyková, 2006). Although the original definition of aurantiactinomyxon also stated that the valvular processes embraced with their whole base the epispore cavity (Janiszewska, 1957), this criterion has been widely disregarded by researchers (see types in Burtle et al., 1991; Bellerud, 1993; Yokoyama et al., 1993; Yokoyama, 1997; Trouillier et al., 1996; Hallett et al., 1997; El-Mansy et al., 1998a, b; Székely et al., 1998; Özer et al., 2002a; Rosser et al., 2014; Milanin et al., 2018), not being included in the updated definition by Lom & Dyková (2006).

Recently, Rocha et al. (2019a) showed that the shape of the valvular processes is a morphological character too variable for distinguishing between raabeia and echinactinomyxon, based on the observation of a type producing actinospores with long valvular processes that were either curved or straight. Consequently, the demise of the echinactinomyxon collective group was proposed and the definition of raabeia was updated to encompass actinospores having straight valvular processes (Rocha et al., 2019a). Similarly, many of the aurantiactinomyxon types included in this synopsis display ambiguous features allowing conformity with the definitions of other collective groups. For instance, Aurantiactinomyxon janiszewskai and the Aurantiactinomyxon type of Xi et al., 2013 have long valvular processes that best resemble those of raabeia (Bellerud, 1993; Xi et al., 2013). Although the processes of the first curve downwards as traditionally described for aurantiactinomyxon, the second is depicted has having straight processes and would probably be better allocated to raabeia. The boundary between these collective groups is further blurred by the report of aberrant spores displaying unequal and different-shaped caudal processes, as is the case of the Raabeia type 4 of Özer et al., 2002 (see Özer & Wootten, 2002). The distinction between aurantiactinomyxon and neoactinomyxum is also tenuous, given that several aurantiactinomyxon types have short valvular processes, which only differ from neoactinomyxum by being triangular or rounded with slightly pointed ends rather than completely spherical (see types in Hallett et al., 1997; Negredo & Mulcahy, 2001; Oumona et al., 2003; Székely et al., 2000, 2003, 2004; Xi et al., 2015; Zhao et al., 2016). It should be noted that a few neoactinomyxum have been reported to have triangular valvular processes [see types described by Borkhanuddin et al. (2014) and Xi et al. (2015)].

However, it is with guyenotia that the lack of a distinctive boundary is most evident. Several aurantiactinomyxon types described in the literature have digitiform valvular processes that conform with the definition of guyenotia, including the Aurantiactinomyxon type of Burtle et al., 1991, Aurantiactinomyxon type of El-Matbouli et al., 1992, Aurantiactinomyxon type 1 of Yokoyama et al., 1993, Aurantiactinomyxon type 3 of Hallett et al., 1997, Aurantiactinomyxon types 2 and 5 of El-Mansy et al., 1989a, Aurantiactinomyxon type 3 of Özer et al., 2002, Aurantiactinomyxon type 1 of Oumouna et al., 2003, Aurantiactinomyxon type A of Eszterbauer et al., 2006, and the Aurantiactinomyxon of Hallett et al., 2006. The inclusion of types with digitiform processes within the aurantiactinomyxon collective group was previously noticed by Xiao & Desser (1998), who suggested they should be transferred to guyenotia. However, the fallibility of this morphological criterion has led authors to compare aurantiactinomyxon and guyenotia interchangeably (see Burtle et al., 1991; Eszterbauer et al., 2006; Xi et al., 2013). Moreover, Hallett et al. (2002) proved that a single aurantiactinomyxon type can produce actinospores with different process length and shape, having observed two distinct phenotypes associated with the same genotype: one displaying swollen, leaf-like processes with either pointed or rounded ends, and the other having elongated, digitiform-like processes. This clearly shows that there is no real boundary between aurantiactinomyxon and guyenotia. Consequently, the demise of the guyenotia collective group is here proposed, with the transference of its types to aurantiactinomyxon. Original names are retained so as not to increase confusion. The decision to invalidate the oldest group rather than the most recent one relates to the low number of guyenotia that have been reported in the literature; only 5 types of guyenotia have been described versus the 61 types of aurantiactinomyxon that are presently known (see Table 1). Accordingly, aurantiactinomyxon is tentatively defined as having a spherical, subspherical, cylindrical or triangular actinospore body with 3 polar capsules protruding from the apex. Three equally sized latero-posterior valvular processes arise from the actinospore body without a style, curving downwards and tapering to a rounded or pointed end, being leaf-like, propeller-like, digitiform or triangular. Nonetheless, this should be regarded as a temporary definition, given that the increase of our knowledge of actinospore biodiversity will undoubtedly blur even more the boundaries between aurantiactinomyxon, raabeia, and even neoactinomyxum. Overall, this “continuum of form” demonstrates that a general shift is needed in our approach to actinospore grouping (Atkinson, 2011), which should probably be based on actinospore functionality relative to environment and host ecology, rather than on morphology.

The great majority of aurantiactinomyxon types reported in the literature infect freshwater oligochaetes belonging to the family Naididae Ehrenberg, 1828 [currently includes members of the former Tubificidae (Erséus et al., 2008)], with reports mainly from the species *Branchiura sowerbyi* Beddard, but also *T. tubifex*, *Limnodrilus hoffmeisteri* Claparède, and *Dero digitata* (Müller), and less frequently from *Lophochaeta ignota* Štolc, and members of the genera *Nais* Müller and *Pristina* Ehrenberg. A few types have their oligochaete hosts identified only up to the genus- or family-level (see Marques, 1984; Grossheider & Körting, 1992; Benajiba & Marques, 1993), while a few others lack host information (see El-Mansy et al., 1998a; Oumouna et al., 2003; Hallett et al., 2006). Only the three Aurantiactinomyxon types described by Hallett et al. (1997), and the Aurantiactinomyxon type of Rocha et al., 2019, are known to occur in the marine environment, parasitizing naidid oligochaetes belonging to the genera *Limnodriloides* Pierantoni, *Pacifidrilus* Erséus, and *Tubificoides* Lastočkin. The only exceptions to the usage of naidids as hosts are Aurantiactinomyxon pavinsis, widely reported from the freshwater lumbriculid *Stylodrilus heringianus* Claparède (see Marques, 1984; Oumouna et al., 2003; Holzer et al., 2004; Marcucci et al., 2009), the Aurantiactinomyxon of Freeman & Kristmundsson, 2018, and the Aurantiactinomyxon type of McGeorge et al., 1997 as reported from *Lumbriculus variegatus* (Müller) by Özer & Wootten (2001). The former Guyenotia type of Xiao & Desser, 1998 was also reported from *L. variegatus* (Xiao & Desser, 1998). A few types have been reported from more than a single host species: Aurantiactinomyxon raabei junioris, Aurantiactinomyxon minor, Aurantiactinomyxon of El-Matbouli et al., 1992, Aurantiactinomyxon of Benajiba & Marques, 1993, and Aurantiactinomyxon of Székely et al., 1998 supposedly infect more than a single naidid species (Table 1), while Aurantiactinomyxon pavinsis and the Aurantiactinomyxon type of McGeorge et al., 1997 have been reported from both naidids and lumbriculids (Table 1). Considering that these reports are not backed-up by molecular data, Rocha et al. (2019c) suggested that aurantiactinomyxon might be host specific, further proposing that actinospores of new isolates be identified through a comprehensive morphological and biological comparison to known types sharing the same annelid host.

Individual prevalence of infection of aurantiactinomyxon types is typically low, ranging from 0.01% to 1.5% in wild environments, and from 0.26% to 4.6% in surveys performed from fish farms, though there is evidence of significant spatial and temporal variations (see El-Mansy et al., 1998a,b; Özer et al., 2002b; Eszterbauer et al., 2006) that probably reflect host genetics, proximity, and habitat preferences, as well as abiotic factors (see Alexander et al., 2015 and references therein). Higher prevalence of infection has been reported when considering the number of infected individuals within only a specific host species, rather than in relation to the annelids population that was sampled (see Székely et al., 2000; Negredo & Mulcahy, 2001), or when pooling all aurantiactinomyxon types occurring in a single annelid species to determine the prevalence of infection of the collective group in a specific sampling site (see El-Mansy et al., 1998a,b). Experimental transmission studies have also reported higher values of prevalence of infection. For instance, Székely et al. (1998) reported 12.5% and 16.7% prevalence of infection of the aurantiactinomyxon counterparts of *Thelohanellus nikolskii* Achmerov, 1955 and *Thelohanellus hovorkai* Achmerov, 1964, respectively.

About 60 myxosporean life cycles have been elucidated to date (see Eszterbauer et al., 2015), with aurantiactinomyxon types being actinospore counterparts to *Chloromyxum truttae* (Léger, 1906), *Henneguya exilis* (Kudo, 1929), the PGD agent *Henneguya ictaluri* Pote, Hanson, & Shivaji, 2000, *Henneguya mississippiensis* Rosser et al., 2005, *Hoferellus carassii* Achmerov, 1960, *Hoferellus cyprini* (Doflein, 1898) Berg, 1898, *Myxobolus intimus* Zaika, 1965, *Paramyxidium giardi* (Cépède, 1906) Freeman & Kristmundsson, 2018, *T. hovorkai*, *Thelohanellus kitauei* Egusa & Nakajima, 1981, *T. nikolskii*, and *Thelohanellus testudineus* Liu et al., 2013 (Eszterbauer et al., 2015 and references therein; Zhao et al., 2016, 2017; Rocha et al., 2019c; Borzák et al., 2021). The former Guyenotia type of Eszterbauer et al., 2006 has also been linked to an unidentified *Zschokkella* sp. from *Carassius auratus* Linnaeus, 1758 (Eszterbauer et al., 2006; data in GenBank). Clarification of the life cycles of *H. carassii* and *H. cyprini* were based solely on experimental transmission studies, with all others established through molecular inference, based on DNA match between myxosporean and actinosporean counterparts (99.2% to 100% similarity reported in the literature). However, the 18S rDNA sequences of the actinospores reported to match *H. ictaluri* and *H. exilis* were not made available (see Lin et al., 1999; Rosser et al., 2014), so that molecular information can only be found for the myxosporean stage. In turn, no sequence is available for the myxosporean stage of *T. hovorkai*, which accounts for two distinct actinospore stage sequences in GenBank. Anderson et al. (2000) reported a single 710 bp 18S rDNA sequence (AJ133419) obtained from both myxosporean and actinosporean stages of *T. hovorkai*. Actinospores were retrieved from infections in *B. sowerbyi* and were identified by the authors as belonging to the Aurantiactinomyxon type 2 of Yokoyama et al., 1993, previously reported to be the life cycle counterpart of *T. hovorkai* based on experimental transmission (see Yokoyama et al., 1993; Yokoyama, 1997; Székely et al., 1998). Later, Eszterbauer et al. (2006) obtained two similarly sized sequences (DQ231153 with 817 bp and DQ231154 with 785 bp) from aurantiactinomyxon actinospores in *B. sowerbyi* that were reported to match unpublished sequences of *T. hovorkai* obtained by the authors during a previous experimental infection study, though being morphologically and genetically different from the Aurantiactinomyxon type 2 of Yokoyama et al., 1993 (see Yokoyama, 1997; Yokoyama et al., 1993; Székely et al., 1998). Presently, both these aurantiactinomyxon types remain identified as life cycle counterparts of *T. hovorkai*, being included as such in Table 1.

A more comprehensive and clear understanding of the diversity of this collective group is necessary to help clarify important interactions with annelid hosts and involvement in myxozoan life cycles. The description of novel types and re-description of known types that remain without molecular data, namely those comprising reports from several hosts, will surely contribute towards this aim. Thus far, molecular-based studies are limited by the paucity of available data but have shown that morphologically similar aurantiactinomyxon actinospores may be distantly related (Rocha et al., 2019c), in the same manner that morphologically different actinospores can share the same genotype (see Hallett et al., 2002; Eszterbauer et al., 2006; Zhao et al., 2016). Consequently, the combined analysis of biological, morphological, and molecular criteria is imperative for performing reliable type identification (Rocha et al., 2019c). This task is significantly hampered by the difficulty in obtaining earlier reports, and due to imprecision and confusion of information in the literature.

In this study, a comprehensive summary of the biological characters and morphometry of all types described within the aurantiactinomyxon group and former guyenotia is provided as an important tool for researchers working in this field. Sixty-six types were counted, with data from original descriptions and subsequent reports. Aurantiactinomyxon eiseniellae Ormières & Frézil, 1969 was not included in the count, as Marques (1984) transferred this type to the neoactinomyxum collective group. Morphometric characters include actinospore body length and width, length and width of valvular processes, length and width of polar capsules, and number of secondary cells. Number of coils of polar tubules was not included, given that this information is available only for the Aurantiactinomyxon of Székely et al., 1998 (3–4), Aurantiactinomyxon of Xiao & Desser, 1998 (3–4), Aurantiactinomyxon of Rocha et al., 2019c (4–5), and the Guyenotia of Xiao & Desser, 1998 (3–4) (Borzák et al., 2021; Rocha et al., 2019c; Székely et al., 1998; Xiao & Desser, 1998). Information on host, locality and availability of molecular data is also provided.
